# Methane Bubbled Through Seawater Can be Converted to Methanol With High Efficiency

**DOI:** 10.1002/advs.202412246

**Published:** 2025-01-21

**Authors:** Xiaowei Song, Chanbasha Basheer, Jinheng Xu, Muhammad Mustapha Adam, Richard N. Zare

**Affiliations:** ^1^ Department of Chemistry Stanford University 380 Roth Way Stanford, CA 94305 USA; ^2^ Chemistry Department King Fahd University of Petroleum and Minerals Academic Belt Road Dhahran 31261 Saudi Arabia

**Keywords:** air‐water interface, alternating potential, methane oxidation, microbubbles, reactive oxygen species

## Abstract

Partial oxidation of methane (POM) is achieved by forming air‐methane microbubbles in saltwater to which an alternating electric field is applied using a copper oxide foam electrode. The solubility of methane is increased by putting it in contact with water containing dissolved KCl or NaCl (3%). Being fully dispersed as microbubbles (20–40 µm in diameter), methane reacts more fully with hydroxyl radicals (OH·) at the gas‐water interface. The alternating voltage (100 mV) generates two synergistic POM processes dominated by Cl^−^ → Cl· + e^−^ and O_2_ + e^−^ → O_2_^−•^ under positive and negative potentials, respectively. By tuning the frequency and amplitude, the extent and path of the POM process can be precisely controlled so that more than 90% methanol is selectively formed compared to the two byproducts, dichloromethane, and acetic acid. The methane to methanol conversion yield is estimated to be 57% at a rate of approximately 887 µM h^−1^. This method appears to have potential for removing methane from air using seawater or for converting higher‐concentration methane sources into value‐added methanol.

## Graphical abstract

Methane gas in microbubbles can be effectively oxidized to methanol by applying low alternating current to a copper oxide mesh electrode soaked in saltwater. With the methane conversion yield over 50%, the conversion rate is over 887 µM h^−1^ and methane‐to‐methanol selectivity over 90%.

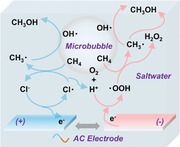

## 1 Introduction

As the major component of natural gas, methane (CH_4_) is emitted in huge amounts annually from various sources, including wetlands, coal mining, agriculture, and oil and gas systems. It is a greenhouse gas that has a 25‐fold higher thermal potency than carbon dioxide (CO_2_) and accounts for approximately 30% of global temperature rise since the Industrial Revolution.^[^ ^1^, ^2^ ^]^ Partial oxidation of methane‐to‐methanol (POM) conversion offers a viable way to cut greenhouse gas leaks and emissions while producing more value‐added products.^[^ ^3^ ^]^ The production and composition of syngas impose constraints on the two‐step process used in traditional conversion methods, which results in poorer methanol yields and higher carbon emissions.^[^ ^4^, ^5^ ^]^ This method is also inappropriate for green and economic methane usage because it operates under high‐pressure and high‐temperature conditions.^[^ ^6^, ^7^ ^]^

The C─H activation of CH_4_ is one of the “holy grails” in the catalysis field because this molecule has a nonpolar tetrahedral shape and strong C─H bonding energy of 439 kJ mol^−1^.^[^ ^8^, ^9^ ^]^ From another aspect, methane oxidation can also be easily overdone to form other oxygenates such as methyl peroxide, formic acid, acetic acid, and CO_2_ because its target POM product, methanol, is much more easily oxidized than CH_4_.^[^ ^10^, ^11^, ^12^ ^]^ This fact poses another challenge for sophisticated control over the POM path.

A wide range of catalysis strategies have been extensively developed including thermal catalysis,^[^ ^13^, ^14^, ^15^ ^]^ photocatalysis,^[^ ^16^, ^17^, ^18^, ^19^, ^20^ ^]^ and electrochemical oxidation.^[^ ^21^, ^22^, ^23^, ^24^ ^]^ Thermal catalysis employs transition metals like palladium, gold, and copper dispersed on porous supporting material like alumina, zeolite, or a metal organic framework to increase surface area and activity. The high temperature and catalyst poisoning with the operation time remain the major issue of this strategy. Photocatalysis is a promising alternative that uses photosensitive semiconductors loaded with various metal alloys to promote C─H activation at room temperature. However, it also faces practical challenges in low solar energy conversion efficiency, high recombination rates of photogenerated carriers, complex catalyst engineering strategies, expensive oxidants like hydrogen peroxide (H_2_O_2_). In contrast, electrocatalytic oxidation could be another promising resort because the interfacial charge transfer‐based redox process can be more precisely and digitally controlled. The charge separation of electron (e^−^) and hole (h^+^) by contact electrification across water‐air‐solid interfaces have been proved to be an effective way to generate reactive oxygen species such as hydroxyl radicals (OH·) and hydrogen peroxide (H_2_O_2_).^[^ ^24^ ^]^

The presence OH· and H_2_O_2_ at the water microdroplet interface has been increasingly supported by both experimental observations and theoretical studies.^[^ ^25^, ^26^, ^27^, ^28^, ^29^, ^30^, ^31^ ^]^ In our previous work, we demonstrated that spraying water microdroplets can facilitate the oxidation of methane to methanol in the presence of atmospheric oxygen and ultrasound.^[^ ^32^ ^]^ Various reactive oxygen species at the gas‐water interface (GWI) are crucial in the partial oxidation of methane (POM). However, the spraying process occurs rapidly, on the millisecond timescale, allowing only a small fraction of methane molecules to interact with the reactive GWI region, while a significant portion of methane remains unreacted. Furthermore, achieving precise control over the POM to avoid overoxidation remains a significant challenge. This underscores the need for improvements in methanol selectivity and conversion efficiency.

As the inverse of water microdroplets, micron‐sized bubbles are likely to exhibit similar GWI characteristics. Microbubbles have been employed in water treatment processes due to their high concentrations of reactive oxygen species at the GWI.^[^ ^33^, ^34^, ^35^ ^]^ This insight led us to consider replacing the spraying microdroplet approach with a microbubbling system to enhance the overall methane conversion yield. Microbubbles are a promising alternative because they tend to persist in water longer before shrinking, coalescing, or bursting.^[^ ^36^ ^]^ Consequently, the extended lifespan of gas substrates within microbubbles offers a prolonged reaction time, potentially improving the conversion of methane into oxygenated products. Our most recent works not only proved the existence of H_2_O_2_ in the electrogenerated microbubble interface but also showed its application in C─H activation and methane oxidation.^[^ ^37^, ^38^ ^]^

Inspired by these rationales, we are motivated to develop an environment‐friendly and economical microbubble interfacial electrochemical oxidation method to scale up the methane removal from air and its conversion to methanol in a highly selective way. Micro‐bubbling integrated with electrocatalysis under alternating potential strategy is presented. The use of potassium chloride and sodium chloride in the developed system are explored with the expectation that seawater, as a widely available natural resource, can be used in this processing method.

## 2 Results and Discussion

### 2.1 Construction of POM Setup

**Figure 1:**
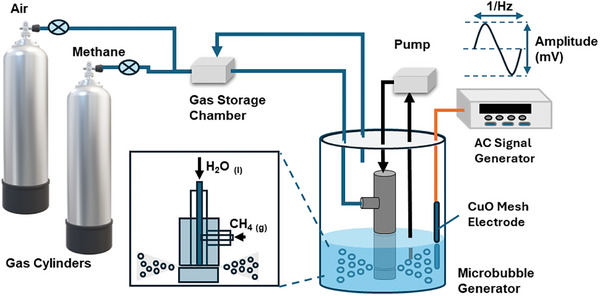
Diagram of the scaleup setup for the methane removal. The major components include compression gas cylinders and regulators, check valves, a closed chamber for gas storage and feeding, a water circulation pump, a microbubble generator, the reaction beaker with saltwater, an AC function generator, a CuO mesh electrode, and Teflon tubing. The inset shows the detailed configuration of the microbubble generator probe.

We developed an initial prototype setup designed for methane removal from air, with its critical components illustrated in **Figure** **1**. Methane and air are supplied through two compressed gas cylinders, with the mixing ratio adjustable to simulate realistic atmospheric methane concentrations by regulating the partial pressures and flow rates. The primary component is a microbubble generator submerged in saltwater that simulates seawater. A circulation pump (1 L min^−1^, 87 psi) injects water into a narrow vertical channel within the microbubble generator probe (inset in Figure 1). This action creates a localized low‐pressure zone within the probe chamber, which draws the air‐methane mixture into a horizontal channel. At the junction spacer, the gas and water fully mix before being expelled through the probe outlet, generating microbubbles. The clear saltwater assumes a white, emulsion‐like appearance during operation (Figure S1, Supporting Information), with microbubbles ranging narrowly in size from 20 to 40 µm. Unreacted gas within the microbubbles rises to the water surface after traveling from the bottom of the reaction container (100 mm in height) and is recaptured by the probe for subsequent oxidation cycles.

The second core part of the experimental setup is the electrocatalysis system, composed of an AC waveform function generator connected to the copper oxide (CuO) mesh electrode by a conducting wire. The CuO mesh has an average pore size of 200 µm to ensure sufficient gas diffusion and mass/electron transfer between the gas‐liquid‐solid tri‐phases.

### 2.2 POM Products Detection

**Figure 2:**
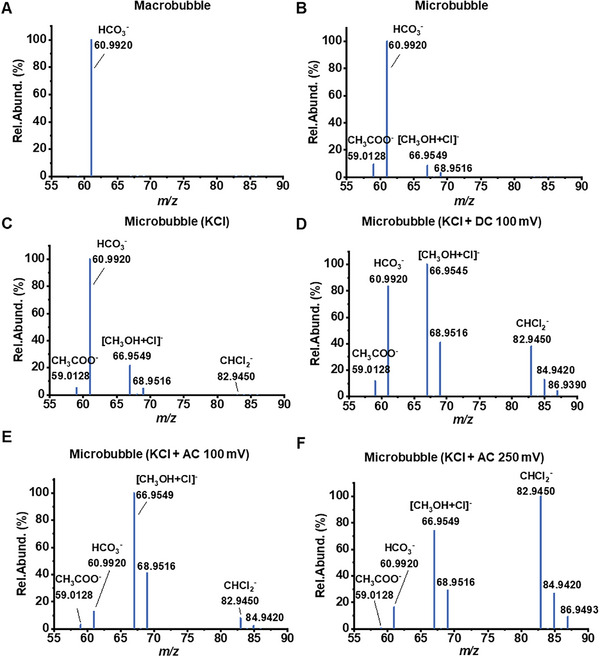
Representative mass spectra of water samples that were bubbled with methane gas under different conditions. a) methane gas pumped into deionized water to generate microbubbles; b) disperse methane gas into DI water to form micron‐size bubbles; c) disperse methane gas in saltwater (KCl, 3%) to form micron‐size bubbles; d) application of a +100 mV direct current (DC) potential onto a copper oxide mesh electrode inserted into the beaker containing methane microbubbles and saltwater; e) application of an alternating current (AC) potential (amplitude ± 100 mV, frequency 50 Hz) onto a copper oxide mesh electrode inserted into the beaker containing methane microbubbles and saltwater; f) same as (e) except the AC potential parameters are amplitude ± 250 mV and frequency 50 Hz.

The experiment commenced by directly injecting the methane‐air gas mixture into deionized water as a negative control. Methane gas was continuously pumped into the water, forming large bubbles with diameters exceeding 1 mm. After one hour of sparging, the sample was analyzed using nanoelectrospray ionization mass spectrometry (nESI‐MS). No ions associated with methanol were detected, with the exception that the bicarbonate ion (HCO_3_^−^, *m/z* 60.9920, **Figure** **2**A) appeared, which likely originated from CO_2_ naturally dissolved in the water. We then used the microbubble generator to pump the methane into the water. Surprisingly, peaks at *m/z* 66.9549 and *m/z* 68.9516 with approximately a 3:1 ratio appeared, and these peaks matched the methanol molecule adducted with a chloride ion ([CH_3_OH + Cl]^−^) based on the mass shift and the abundance of chlorine isotopes. Apart from methanol, an acetic acid peak (HAc, CH_3_COO^−^, *m/z* 59.0128) was detected with an equivalent intensity (Figure 2B). Technically, this result indicated that the trace level of background chloride ions found in the water container and tubing can assist in the ionization of methanol by H‐Cl^−^ hydrogen bonding. More importantly, it agrees with our hypothesis that water microbubbles do have the ability to oxidize methane because of the existence of HO· and H_2_O_2_ across the GWI region, like what we previously discovered at the microdroplet interface.

Recognizing the role of chloride in successfully detecting methanol, we intentionally added potassium chloride to the water, creating a 3% saltwater solution for the microbubbling test. The relative abundance of the methanol peak increased by 20% compared to the unsalted condition. Additionally, a series of weak peaks appeared at *m/z* 82.9450, 83.9430, 84.9420, 85.9454, and 86.9493 (Figure 2C), with distributions matching deprotonated dichloromethane (DCM), namely, CHCl_2_^−^. This result suggests that chloride not only aids in ionization but also participates in the methane oxidation process. Next, we applied a +100 mV DC potential to the CuO mesh electrode (50 mm × 50 mm, 5 mm thickness) immersed in 3% saltwater while microbubbling methane for one hour. The intensity of the methanol ion became comparable to that of the HCO_3_^−^ base peak, while the CHCl_2_^−^ ion intensity increased to 40%, and CH_3_COO^−^ reached 15% (Figure 2D). These results indicate that the application of positive DC potential significantly enhances methane oxidation and chlorination.

Finally, when we replaced the DC potential with a sine waveform AC potential at a frequency of 100 Hz and an amplitude of ±100 mV, the methanol ion intensity surpassed that of HCO_3_^−^, becoming the predominant peak in the mass spectrum. In contrast, the CHCl_2_^−^ and CH_3_COO^−^ peaks did not show significant increases (Figure 2E). This suggests that an AC potential improves methanol selectivity compared to a DC potential. However, when the amplitude was increased from ± 100 mV to ± 250 mV, CHCl_2_^−^ became the base peak instead of methanol (Figure 2F), indicating that the selectivity between methanol and DCM can be reversed by adjusting the potential.

### 2.3 Alternating Potential Optimization

**Figure 3:**
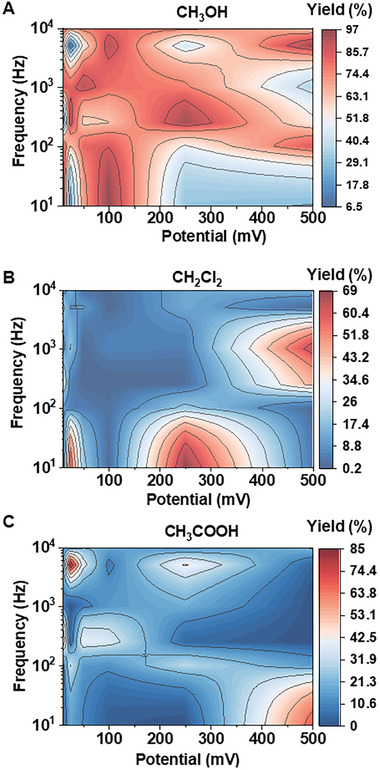
Heatmaps present the influences of the AC frequency and potential on switching the methane removal products among a) methanol, b) dichloromethane, and c) acetic acid.

We systematically optimized the alternating frequency and amplitude to elucidate their roles in influencing methane oxidation pathways and product selectivity. The production of CH_3_OH, CH_2_Cl_2_, and CH_3_COOH was compared under various combinations of frequency, ranging from 10 Hz to 1 MHz, and absolute amplitude, spanning from 10 mV to 500 mV. The selectivity under these conditions is presented as a heatmap (**Figure** **3**A). As shown, the selectivity for methane‐to‐methanol conversion remained consistently high, ranging from 63% to 97%, compared to methane‐to‐dichloromethane and methane‐to‐acetic acid, across multiple frequency and amplitude combinations. Notably, methanol selectivity exceeded 90% under several specific alternating parameter sets, including 10 Hz/±10 mV (94.7%), 10 Hz/±100 mV (96.6%), 250 Hz/±25 mV (90.8%), 250 Hz/±250 mV (94.2%), 1 kHz/±50 mV (91.4%), 5 kHz/±100 mV (91.9%), and 5 kHz/±500 mV (96.2%) (Figure 3A). These findings indicate the existence of multiple local optima for frequencies at different potential values. Additionally, a clear pattern emerged where the optimal potential generally increased with the acceleration of the alternating frequency.

In terms of the CH_2_Cl_2_, its predominant production only showed up in several specific conditions set such as 10 Hz/±25 mV (58.8%), 10 Hz/±250 mV (68.9%) and 1 kHz/±500 mV (64.5%). Even under these conditions, the CH_2_Cl_2_ selectivity remains relatively lower than what can be achieved for the methane‐to‐methanol conversion (Figure 3B). For CH_3_COOH, we found that it is mainly produced on even fewer occasions at 10 Hz/±500 mV (67.8%) and 5 kHz/± 25 mV (85%) (Figure 3C). These results clearly demonstrate that the methane‐to‐methanol conversion can be selectively and efficiently achieved by precisely controlling the alternating potential amplitude and frequency applied to the CuO mesh electrode immersed in saltwater through which methane is microbubbled.

In addition to characterization via nano‐electrospray ionization mass spectrometry (nESI‐MS), the POM products were further validated through nuclear magnetic resonance (NMR) spectroscopy. Three representative samples, prepared under optimal conditions favoring the production of CH₃OH (10 Hz/±100 mV), CH₂Cl₂ (10 Hz/±250 mV), and CH₃COOH (10 Hz/±500 mV), were analyzed using proton NMR (¹H‐NMR). The integration of peaks corresponding to chemical shifts at 5.3 ppm (CH₂Cl₂), 3.3 ppm (CH₃OH), and 2.0 ppm (CH₃COOH) demonstrated an abundance ratio highly consistent with that predicted by ion intensities obtained from mass spectrometry (Figures S2–S4, Supporting Information).

### 2.4 Quantitative Evaluation of the POM Process

Following the investigation of frequency and potential, the combination of 10 Hz and ±100 mV was selected as the optimal condition due to its highest yield and relatively low energy input. The precise concentrations of methanol, dichloromethane (DCM), and acetic acid (HAc) were determined using a set of quantification curves derived from a dilution series of standard solutions for each compound. A linear response was observed between the normalized ion intensity and concentration within the ranges of 100–2000 µM for methanol, 50–1000 µM for DCM, and 10–200 µM for HAc, respectively (Figure S5, Supporting Information).

**Figure 4:**
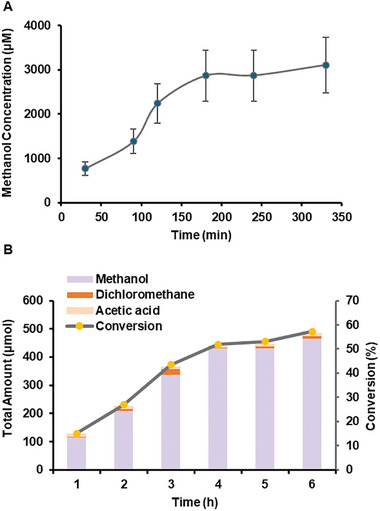
Quantitative estimation of the methane removal performance: A) The concentration of generated methanol with processing time (n = 3); and B) Total amount (left vertical axis) of methane removed from the air and conversion yield (right vertical axis).

The methane‐to‐methanol conversion process was monitored for over 6 hours. As shown in **Figure** **4**A, the methanol concentration gradually increased for the first 3 hours. Thereafter, the methanol concentration reached a relatively constant level of 2948 ± 137 µM. This might be attributed to the depletion of available oxygen in the air and water for the partial methane oxidation process. In contrast, the concentrations of generated DCM and HAc remained at a constant level of 60.5 ± 12.5 µM and 45.4 ± 3.8 µM.

Regarding conversion efficiency, approximately 480 µmol of total carbon was converted by microbubbling methane gas into 150 mL of saltwater over a 6‐hour period (Figure 4B). Considering a 10% methane composition in the total volume of 200 mL of gas consumed (36 mL h^−1^), the total amount of methane molecules was estimated to be approximately 838 µmol, based on the ideal gas law at 101 kPa and 288 K. Consequently, the overall methane conversion yield was determined to be 57.3%, with a methane‐to‐methanol conversion yield of 55.6% and a selectivity as high as 97%. This performance, no matter what is the selectivity, yield, rate, or operation condition, shows highly competitive advantages compared to many known reports to the best of our knowledge (Table S1, Supporting Information).

### 2.5 Capture of Reactive Radicals and Crucial Intermediates

**Figure 5:**
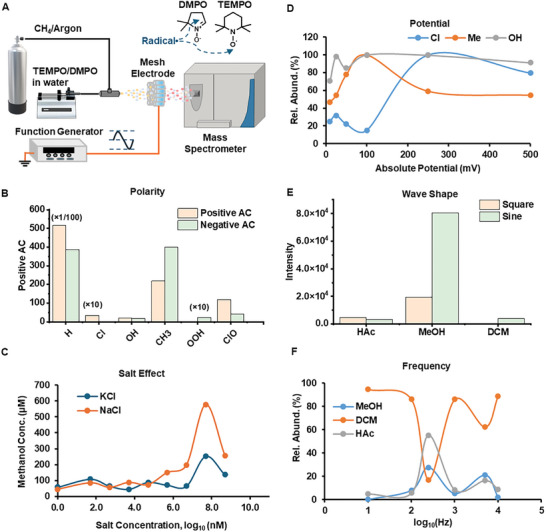
Critical factors influence the generation of radicals and products during the methane removal process. A) Diagram of the on‐line mass spectrometer setup for capturing radicals and intermediates generated by the AC potential during the methane removal process; B) Influence of the salt type and concentration on the methanol generation; C) Influence of the polarity of the AC potential on different radicals and intermediates; D) Abundance changes of three critical radicals as the absolute potential amplitude increases; E) Influence of the wave shape on the three major products; and F) Impact of AC frequency on tuning the switching of methane removal products between methanol and dichloromethane.

We developed an alternative process monitoring setup to gain insights into the POM (partial oxidation of methane) mechanism (**Figure** **5**A). The system utilizes a tee‐union with coaxial capillary sprayers to mix methane and water, generating microdroplets. To mitigate the risk of ignition, methane was premixed with argon (partial pressure ratio of 5:95) rather than air. The water phase contained two spin traps, TEMPO and DMPO, employed to capture reactive radicals and key intermediates.

A CuO mesh electrode, pre‐soaked in 3% KCl solution and thoroughly dried, was connected to an AC function generator and positioned in front of an Orbitrap mass spectrometer (MS) inlet (Figure S6, Supporting Information). As the methane‐water microdroplets passed through the mesh electrode, the dissolved salt became involved in the POM reaction at the gas‐liquid‐solid tri‐interface, closely simulating the conditions in the microbubble system.

Given the known concentrations of TEMPO (6.4 mM) and DMPO (8.9 mM), the concentrations of various radicals were estimated by comparing the ion intensities of reacted species to the unreacted TEMPO or DMPO ions. As a result, TEMPO and DMPO effectively captured several major reactive species: hydrogen radical (H· 1.89 ± 0.21 mM), hydroxyl radical (OH·, 2.60 ± 0.32 µM), and methyl radical (CH_3_·, 9.46 ± 2.87 µM). Additionally, small amounts of chlorine radical (Cl· 0.29 ± 0.22 µM) and hydroperoxyl radical (OOH·, 0.13 ± 0.05 µM) were also detected by the MS. These species were observed under applied potentials ranging from 10 to 500 mV and frequencies from 10 Hz to 1 MHz.

### 2.6 Influence of Salt and Alternating Potential Parameters

We continued exploring other critical factors that could influence radical generation and reaction pathways to further elucidate the mechanism. First, we observed that more H· and Cl· radicals were detected only in the working mode with a positive alternating potential. In contrast, higher concentrations of CH_3_· and OOH· radicals were observed under a negative alternating potential‐only mode. The amount of OH· remained stable between positive and negative potential modes (Figure 5B). These findings suggest that Cl· and OOH· are the predominant species responsible for activating the C─H bond of CH_4_ during the positive and negative phases, respectively. We speculate that Cl· originates from the single‐electron oxidation of Cl⁻ under positive potential (with CuO serving as the anode), while OOH· likely arises from the reduction of O_2_ dissolved in water or air, facilitated by H· (or H⁺ from HCl) and electrons donated by the CuO cathode.

The addition of salt, such as sodium chloride or potassium chloride, is crucial for providing Cl^−^ to generate Cl·, which initiates the POM reaction. This is evidenced by a 12‐fold increase in methanol production when the salt concentration exceeded 50 mM (Figure 5C). The presence of chloride ions also enhances methane's solubility in water caused by weak interactions with the methane molecules.^[^ ^39^, ^40^ ^]^

Upon tuning the applied alternating potential, the abundance of CH_3_· reached its maximum at +100 mV, whereas Cl· peaked at +250 mV. This trend persisted across varying frequencies (10 Hz, 100 Hz, 1 kHz, and 1 MHz) (Figures S7,S8, Supporting Information). These potentials are consistent with prior findings where maximum CH_3_OH production occurred at 10 Hz/±100 mV and CH_2_Cl_2_ production at 10 Hz/±250 mV. Interestingly, the relative abundances of CH_3_· and Cl· appeared competitive; as one increased, the other decreased. Nevertheless, both radicals maintained a stable level of OH·, essential for methane oxidation (Figure 5D). This indicates that two distinct, radical‐driven processes occur independently yet synergistically during the alternating potential cycles. This hypothesis is supported by the observation that a sine waveform alternating potential was more effective than a square waveform (Figure 5E). The smooth transition of a sine wave better coordinated the two synergistic methane oxidation processes, whereas a square wave favored only one oxidation pathway.

Further investigation into alternating frequencies revealed that CH_3_OH and CH_2_Cl_2_ production exhibited complementary, competitive trends. CH_3_OH production dominated at frequencies of 10 Hz (100 ms⁻¹), 100 Hz (10 ms⁻¹), 1 kHz (1 ms⁻¹), and 1 MHz (0.1 ms⁻¹). In contrast, CH_2_Cl_2_ production was favored at 250 Hz (4 ms⁻¹) and 5000 Hz (0.2 ms⁻¹) (Figure 5F). These findings suggest that the CH_3_OH and CH_2_Cl_2_ pathways have distinct reaction kinetics, with CH_3_OH formation occurring on a timescale of approximately 100 µs, whereas CH_2_Cl_2_ formation is slower, around 200 µs. Although Cl· is crucial for initiating C─H activation in CH_4_ to form CH_3_· and HCl, chlorination appears to be slower than methane oxidation, where CH_3_OH is formed from CH_3_· and OH·. This explains why alternating electrocatalysis at the right frequency achieves high selectivity for CH_3_OH over its byproduct, CH_2_Cl_2_.

### 2.7 Possible Reaction Mechanisms

The conversion of methane to methane has been achieved through a complex series of radical‐initiated chemical reactions, and it would seem foolhardy to claim that a full understanding of the mechanism for this process has been achieved. Nevertheless, the experimental findings described above do suggest a broad outline of what happens in the POM process. The alternating redox potential leads to synergistic methane oxidation, composed of two working phases. Under a positive potential, Cl· is the major activation radical, and the CuO mesh electrode serves as an anode to remove an electron from Cl^−^ to generate reactive Cl· (Equation (1)). Cl· reacts with CH_4_ to generate CH_3_· (Equation (2)) and liberate in solution H^+^ and Cl^−^. At the same time, at the GWI, OH^−^ loses an electron to form the OH· radical (Equation (3)), which recombines with CH_3_· to form methanol (Equation (4)). Overall, the sum of these steps is shown in Equation (5).

(1)

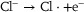

(2) $$\mathrm{Cl}\cdot +CH_{4}\to CH_{3}\cdot +\mathrm{HCl}\;(H^{+}+Cl^{-})$$

(3) $$OH^{-}\to \mathrm{OH}\cdot +e^{-}$$

(4) $$CH_{3}\cdot +\mathrm{OH}\cdot \to CH_{3}\mathrm{OH}$$

(5) $$CH_{4}+OH^{-}\to CH_{3}\mathrm{OH}+H^{+}+2e^{-}$$

Under a negative potential, O_2_ is the major source of activation radical, and the CuO mesh electrode serves as a cathode to donate one electron to generate reactive O_2_^−^ (Equation (6)), which can further attach the H^+^ generated from HCl (Equation (2)) in the positive potential phase to form the hydroperoxide radical OOH· (Equation (7)). The OOH· radical can react with CH_4_ to generate either H_2_O_2_ and CH_3_· (Equation (8)) or CH_3_OH and OH· (Equation (9)). The formed H_2_O_2_ can also be decomposed to 2 OH·, which is catalyzed by CuO in a Fenton‐like reaction (Equation (10)). Once again, OH· recombines with CH_3_· to yield methanol (Equation 11); the overall reaction is shown in Equation (12).

(6) $$O_{2}+e^{-}\to {O_{2}}^{-}$$

(7) $$O_{2}+H^{+}\to \;\mathrm{OOH}\cdot$$

(8) $$\mathrm{OOH}\cdot +CH_{4}\to CH_{3}\cdot \;+\mathrm{HOOH}$$

(9) $$\mathrm{OOH}\cdot +CH_{4}\to CH_{3}\mathrm{OH}+\mathrm{OH}\cdot$$

(10) HOOH→2OH·

(11) $$CH_{3}\cdot \;+\mathrm{OH}\cdot \;\to CH_{3}\mathrm{OH}$$

(12) $$CH_{4}+O_{2}+H_{2}O\to CH_{3}\mathrm{OH}+2\;\mathrm{OH}\cdot$$

The amount of acetic acid suggested that methane tends to be overoxidized in microbubbles as well. Acetic acid can be formed through multiple radical reaction pathways. First, the generated methyl radical (CH_3_·) can react with the CO generated from the methane partial oxidation and OH· across the water microbubble interface. In addition, the methyl radical can also react with the CO_2_ either from the air or the methane overoxidation to form CH_3_COO·, followed by the hydrogenation to form CH_3_COOH or single electron transfer to form CH_3_COO^−^. The third way is that two methyl radicals will form ethane (C_2_H_6_) first and then further be oxidized into acetic acid (Figure S9, Supporting Information).^[^ ^41^, ^42^ ^]^ The gas chromatography (GC) and GC‐MS tests confirmed the existence of approximately 0.3% CO, 0.1% CO_2_, and 0.001% C_2_H_6_ in the gas sample above the microbubbling reaction solution, supporting the three possible paths mentioned above (Figures S10,S11, Supporting Information). In addition, formic acid was also detected in the form of the sodiated dimer [HCOONa+HCOO]^−^ at the *m/z* 112.9845. However, its intensity was only at an ignorable level of E1‐E2, which was much lower than the level of acetic acid (E3), methanol (E4‐E5), and dichloromethane (E3‐E4) (Figure S12, Supporting Information).

During this POM process, the chloride ion can increase the rate of methane conversion into methanol by serving as a catalyst. This ion changes the electrical properties of the metal catalyst and stabilizes reactive intermediates, stopping methanol from being overoxidized and making it easier to form methanol. Additionally, chloride ions can aid in producing active species like copper‐chloride complexes, which are necessary for the efficient oxidation of methane. Chloride ions make electrolytes more conductive, keep active catalytic sites stable in electrochemical systems, and improve the efficiency of the conversion process.^[^ ^43^ ^]^

Alternating current (AC) possesses the unique advantage over direct current (DC) in multiple aspects. Varied potentials can fine tune the multiplex redox process within one working period avoiding metal catalyst deposition and loss in catalytic activity.^[^ ^44^, ^45^, ^46^, ^47^ ^]^ It can also stabilize active species near the electrode surface and minimize mass transfer distances. The AC potential quickly switches a single electrode's working mode between anode and cathode, avoiding metal catalyst deposition and boosting the duration. It provides the necessary energy to overcome activation barriers, coordinate two reaction routes to facilitate them both, and ensure the continuous regeneration of reactive species like Cl·, OH·, and OOH·. It was also worth noting that alternating current can significantly change reaction outcomes by controlling the intensity of oxidation and reduction. Apart from methanol, other value‐added products like acetic acid and dichloromethane can also be intentionally produced by controlling the alternating frequency and potential. This shows the largest advantage of AC over conventional DC in controlling the POM process.

Copper oxide is known to promote the breakdown of C─H bonds in methane and produce active oxygen species through water vapor oxidation.^[^ ^48^, ^49^, ^50^, ^51^, ^52^ ^]^ However, the CuO's efficacy as an electrocatalyst may be hampered by its propensity to be reduced to Cu at potentials higher than its functional potential. This issue might be well resolved by changing the polarity on the CuO mesh throughout the microbubbling process. It also makes a very important contribution to creating a highly reactive gas‐water interface, facilitating methane gas dispersal and extending the methane gas dwell time at the GWI.

## 3 Conclusion

The trade‐off between reactivity and selectivity of direct methane‐to‐methanol conversion is still challenging to balance, particularly at room temperature. Methanol is readily overoxidized into CO and CO_2_, reducing methanol selectivity as methane conversion rises. This study successfully developed a highly selective partial oxidation method to convert methane gas to methanol. This is accomplished by bubbling methane through saltwater, to which a low‐voltage alternating potential is applied to a copper oxide mesh. The total conversion yield is above 57% at a rate of 887 µM h^−1^. The selectivity of methanol is above 90% versus the other products. The method only uses an alternating potential as low as 10 mV. The treatment can directly use saltwater or even seawater to remove the methane emission from the air and convert it into value‐added products. Further work is in progress to scale up this process and determine its possible practicality.

## 4 Experimental Section

### Experimental

More details about the experimental description can be found in the supporting information including reagents and materials, CuO mesh electrode preparation, microbubble‐electrocatalysis reactor for methane oxidation, methane oxidation products detection by nESI‐MS, quantitation of methanol, dichloromethane, and acetic acid, methane oxidation products identification by ^1^H‐nuclear magnetic resonance, on‐line capture of radicals and intermediates by mass spectrometry.

### Statistical Analysis

Mass spectrometry data was saved in *.raw format created by the Thermo Fisher and can be read and analyzed by its commercial software Xcalibur. The ion intensity was normalized by the base peak intensity across the scan range. Data was presented as mean ± standard deviation with at least n = 5 sampling points. Statistical methods were not applied to this work. All the data analysis was conducted in Microsoft excel and the graphs were plotted in the Origin or Microsoft power point.

## Conflict of Interest

The authors declare no conflict of interest.

## Author Contributions

X.S. and C.B. contributed equally to this work. X.S. and R.N.Z. conceptualized the idea of using microbubble interfacial reactive oxygen species for methane removal. C.B. introduced the idea of combining microbubble strategy with electro‐oxidation in saltwater under alternating redox potentials. C.B. and J.X. conducted the electro‐oxidation experiment. X.S. conducted the methane oxidation products characterization and quantitation by mass spectrometry. X.S., C.B., and J.X. work together to construct the microbubbling electro‐oxidation prototype for the methane removal. X.S. and C.B. built up the microdroplet electro‐oxidation setup for the on‐line monitoring of radicals and intermediates. M.M.A conducted the gas chromatography mass spectrometry analysis. X.S. and C.B. drafted the initial manuscript. R.N.Z. monitored the whole work and revised the manuscript.

## Supporting information



Supporting Information

## Data Availability

The data that supports the findings of this study are available from the corresponding author upon reasonable request.
