# Supplementary material for: Methane Bubbled Through Seawater Can be Converted to Methanol With High Efficiency
Source: Adv Sci (Weinh). 2025 Jan 21;12(10):2412246. doi: 10.1002/advs.202412246 (PMC11904936; doi:10.1002/advs.202412246)
Supplement: Supplementary file 1 — Supporting Information [file ADVS-12-2412246-s001.pdf]

ADVANCED SCIENCE
Open Access

Supporting Information

for *Adv. Sci.*, DOI 10.1002/advs.202412246

Methane Bubbled Through Seawater Can be Converted to Methanol With High Efficiency

Xiaowei Song, Chanbasha Basheer, Jinheng Xu, Muhammad Mustapha Adam and Richard N. Zare**

Supporting Information

Methane Bubbled through Seawater can be Converted to Methanol with High Efficiency

Xiaowei Song, Chanbasha Basheer, Jinheng Xu,Muhammad Mustapha Adam, Richard N. Zare **

X.Song, J.Xu, R.N.Zare

Department of Chemistry

Stanford University

380 Roth Way, Stanford, 94305, USA

C.Basheer, M.M.Adam,

Chemistry Department

King Fahd University of Petroleum and Minerals

Academic Belt Road, Dhahran, 31261, Saudi Arabia

E-mail: cbasheer@kfupm.edu.sa; zare@stanford.edu

Table of Contents

Items	Captions	Pages
Experimental	Reagents and Materials; CuO mesh electrode preparation; Microbubble-electrocatalysis reactor for methane oxidation; Methane Oxidation Products detection by nESI-MS; Quantitation of methanol, dichloromethane, and acetic acid; Methane Oxidation Products Identification by ^{1}H-Nuclear Magnetic Resonance; On-line capture of radicals and intermediates by mass spectrometry	S3-S4
Figure S1	Photo of the real setup for methane oxidation by saltwater microbubbles and alternating current electrocatalysis.	S5
Figure S2	^{1}H-NMR identification of methane oxidation products generated under the condition of 10 Hz/±100 mV.	S6
Figure S3	^{1}H-NMR identification of methane oxidation products generated under the condition of 10 Hz/± 250 mV.	S7
Figure S4	^{1}H-NMR identification of methane oxidation products generated under the condition of 10 Hz/± 500 mV.	S8

Figure S5	Quantitation curves for determining the concentration of methanol, dichloromethane, and acetic acid generated from the methane oxidation.	S9
Figure S6	Photos of the (A) on-line setup for the methane oxidation and radicals monitoring by high-resolution mass spectrometer. (B) the zoom-in photo of the on-line reactor composed CuO mesh electrode precoated with salt precipitation and an AC probe and a nebulizing sprayer.	S10
Figure S7	Abundance changes of methyl radical with the applied AC potential under different frequencies.	S11
Figure S8	Abundance changes of chlorine radical with the applied AC potential under different frequencies.	S12
Figure S9	Proposed pathways of the acetic acid formation derived from CO, CO_2, and C_2H_6 respectively.	S13
Figure S10	Gas chromatography detection result for the partial oxidation of 1% methane gas in the air after being micro-bubbled into saltwater applied with ± 100 mV alternating potential at a frequency of 10 Hz.	S14
Figure S11	Gas products detection from partial oxidation of methane by gas chromatograph-mass spectrometry. The components were characterized by (A) total ion chromatogram and (B) average mass spectrum.	S15
Figure S12	Typical mass spectrum of partial oxidation of methane that is micro-bubbled through saltwater under the conditions of ± 100 mV and 10 Hz. The formic acid was detected in a sodiated dimer form with trace amount.	S16
Table S1	Performance comparison with different catalytic oxidation of methane into methanol.	S17
References	References	S18

Experimental Section

Reagents and Materials

The laboratory tap water was directly used as a solvent for generating microbubbles. Methanol (>99.9%, HPLC grade, Fisher chemical), dichloromethane (anhydrous, 99.9%, ACROS), acetic acid (≥99.7%, glacial, Fisher Bioreagents), and dimethyl sulfoxide (DMSO ≥99.7%, Fisher Bioreagents) were used as standard for quantitation purpose. Sodium chloride (99.0%, Fisher Chemical) and potassium chloride (99.0%, Fisher Chemical) were added to water for simulating seawater. Two spin trappers, (2,2,6,6-Tetramethylpiperidin-1-yl) oxyl (TEMPO) and 2,2-Dimethyl-3,4-dihydro-2H pyrrole 1-oxide (DMPO) were purchased from Aldrich (95%, Lot #BCBW3928) and Abcam (AB144610, density: 1.015 g/mL), respectively. The compressed air (Prospec UN1002, ultra zero grade), methane (99.97% UHP T, Linde) and argon (UN1066, Grade 5.0, Medipure, Linde) were ordered from Praxair, Inc. (Danbury, CT, USA). The alternating current wave function generator (33220A, 20 MHz function/arbitrary waveform generator, Agilent) and a direct current power supply (TackLife, MDC02) were used to introduce the AC and DC potential for electrocatalysis, respectively. The microbubble generator was provided by YLEC consultants fluid mechanics (Mode: CARMIN D1 single, France). The miniature electric diaphragm pump was ordered from IEIK (Model W0442, DC 12 V, 3 L/min).

CuO mesh electrode preparation

Copper mesh supporter was ordered from TMAXCN (Xiamen, China) and cut into replicates of small pieces (loading area: 5.0 × 5.0 cm^2, thickness 1.6 mm). To prepare, each piece of copper mesh was baked in an autoclave under 400 °C for 6 hours and then gradually cooled to room temperature before use.

Microbubble-electrocatalysis reactor for methane oxidation

The prototype setup for methane oxidation was mainly composed of several parts that are described in what follows: (1) gas supply system, which includes compression gas cylinders, regulators, check valves, and tee-union mixer; (2) water circulation and micro-bubbling system, which includes Teflon tubings, microbubble generator probe, a water circulating pump, and a reaction beaker containing saltwater; and (3) electrocatalytic processing system, which include the AC function generator, copper oxide mesh electrode, conducting wires, and clippers for grounding and connecting with the CuO electrode.

The circulating pump operated at the speed of 1 L/min under a pressure of 87 psi. The optimal salt (NaCl or KCl) concentration was maintained at 500 mM level. The total saltwater volume was 150 mL,at a very slow rate of approximately 36 mL/hour. The microbubble size is narrowly distributed within the range of 20-40 μm. To investigate the optimal combination of AC frequency and potential amplitude, the AC frequency was set at a value between 10 Hz, 100 Hz, 500 Hz, 1 kHz, 5 kHz, and 1MHz. The absolute amplitude of an AC or DC potential was set at a value among 10 mV, 25 mV, 50 mV, 100 mV, 250 mV, and 500 mV. The reaction lasted for 30 minutes for each condition.

Methane Oxidation Products detection by nESI-MS

An orbitrap mass spectrometer (LTQ XL Orbitrap, Thermo Fisher, San Jose, CA, USA) was employed to detect methanol and other methane oxidation products, respectively. Samples were introduced into an MS system by the nano-electrospray ionization (nESI). Briefly, a 10 μL sample solution was first loaded into a 3.5 cm length borosilicate glass capillary from the open end (ID: 1.1 mm) to the tapering end (ID: 3 μm), which was fabricated by a flaming/brown micropipette puller (P-87, Sutter instrument, Novato, CA, USA). When the capillary was

mounted at a distance of 2 mm in front of the MS inlet, a conductive needle was inserted into the capillary from its open end and contacted with the liquid solution inside. Then, a -1.5 kV high voltage was applied to the needle to trigger the spray ionization for the MS data recording. A full scan mode was used within a range of m/z 55 to 100 for the methanol detection. The target ions were *m/z* 59.0128 for acetic acid (CH_3COO^-), 66.9592 and 68.9563 for methanol ($[CH_3OH+Cl]^-$), 82.9450, 84.9420 and 86.9391 for dichloromethane ($[CHCl_2^-]$).

Quantitation of methanol, dichloromethane, and acetic acid

The methanol, dichloromethane, and acetic acid standards were first diluted with water to form 200 mM stock solutions, respectively. Thereafter, for each product standard, a series of diluted solutions were prepared as follows: (1) Methanol: 100, 250, 500, 1000, 2000 μM; (2) Dichloromethane: 50, 100, 250, 500, 1000 μM; (3) Acetic acid: 10, 25, 50, 100, 200 μM. Each concentration solution was directly injected into the Orbitrap MS system by the nESI method. The intensities of *m/z* 66.9945, *m/z* 59.0182, and *m/z* 82.9450 were used to represent the signal responses to the corresponding solution of methanol, dichloromethane, and acetic acid, respectively. The calibration curve was plotted by fitting the average intensity versus the concentration.

Methane Oxidation Products Identification by ^{1}H-Nuclear Magnetic Resonance

The methane oxidation products generated under varied conditions were identified using the nuclear magnetic resonance instrument (Bruker), operating at a proton frequency of 600 MHz and equipped with a digital autotune probe. A 300 μL deuterated water containing DMSO (as internal standard) was mixed with 700 μL sample solution for the ^{1}H-NMR test. The field locking, auto shimming, and manual tuning experiments were conducted before the formal ^{1}H data acquisition to achieve an optimal signal-to-noise ratio condition. Water suppression was also done to further enhance the sensitivity of the measurement. The ^{1}H-NMR peak annotation and peak integration were conducted by MestReNova.

On-line capture of radicals and intermediates by mass spectrometry

A coaxial sonic spray system consisted of a syringe pump (Masterflex, Cole Parmer, Vernon Hills, IL, USA), a silica capillary (ID:200 μm, OD 350 μm, Polymicro Technologies, Phoenix, AZ, USA), stainless steel tubing (ID: 500 μm, OD: 2 mm, Length: 30 mm), and stainless-steel tee (Swagelok, Solon, OH, USA), and compression gas cylinder (Praxair, San Jose, CA, USA). Methane gas and argon were pumped from compression cylinders and mixed under various ratios (5:95). Check valves were connected in the tubing and the mixer to avoid backward flow. A syringe pump was employed to transport the liquid water (HPLC grade, Merck, 10 μL/min) to the nozzle spray outlet. The gas mixture under high pressure (100 psi) then nebulized the bulk water into numerous small microdroplets (10-30 μm average diameter). For the methane oxidation intermediate capture experiment, both DMPO and TEMPO were spiked in water for microdroplets spraying (1 mg/mL). The total reaction time was 1 minute for each AC condition.

Safety Precautions

Even though the reactions were conducted at room temperature, it is essential to note that mixtures of methane and air can potentially be explosive. Hence, it is crucial to ensure that all glassware and equipment used in these reactions can withstand pressure. They should have appropriate containment measures and protective screening to prevent potential hazards. In terms of methane gas, the partial pressure should be no larger than 5% of the argon pressure.

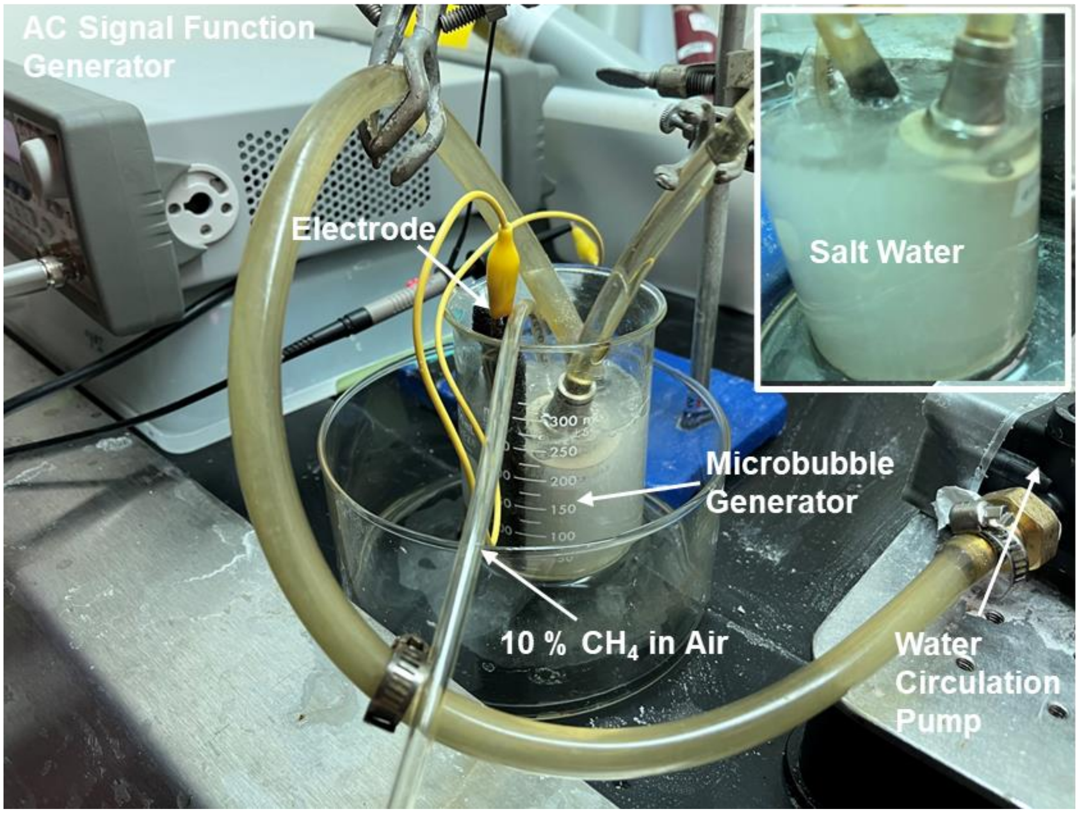

Figure S1. Photo of the real setup for methane oxidation by saltwater microbubbles and alternating current electrocatalysis.

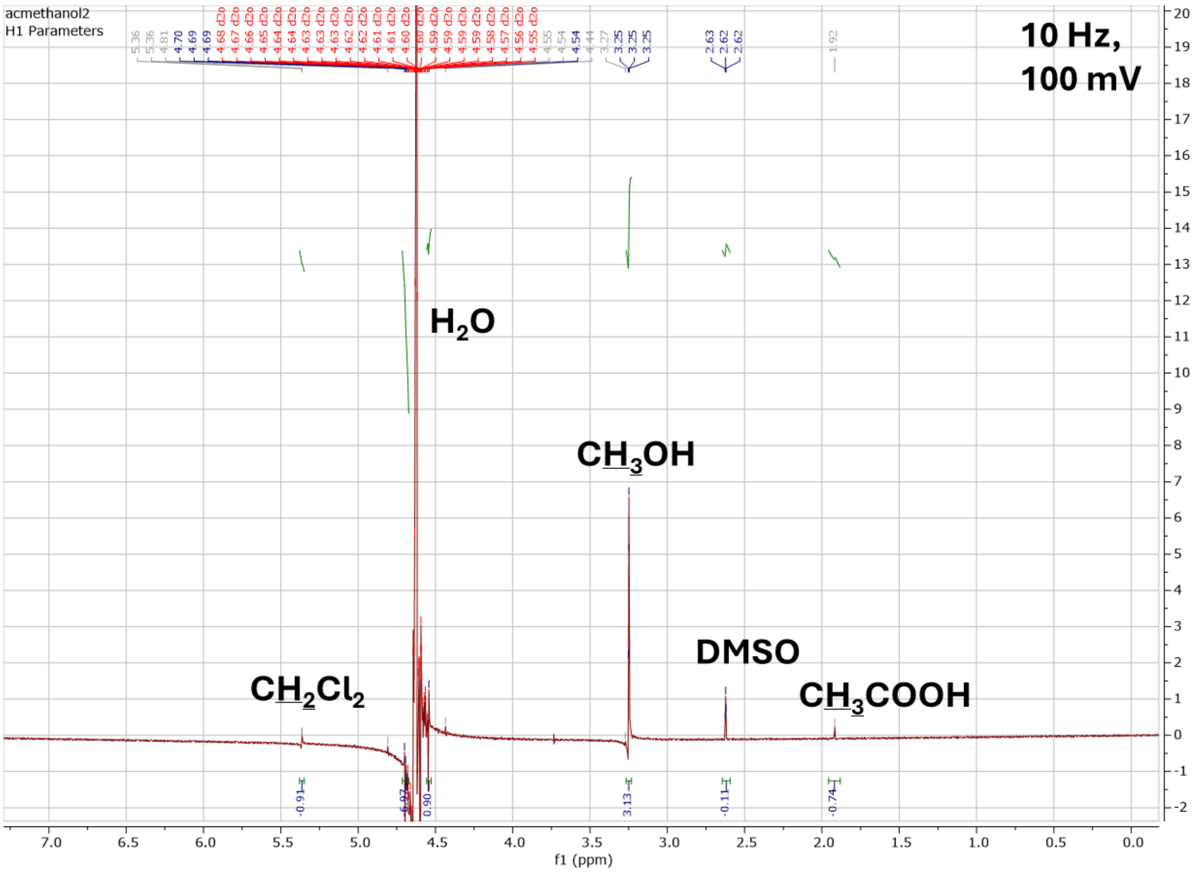

Figure S2. ^{1}H-NMR identification of methane oxidation products generated under the condition of 10 Hz/±100 mV.

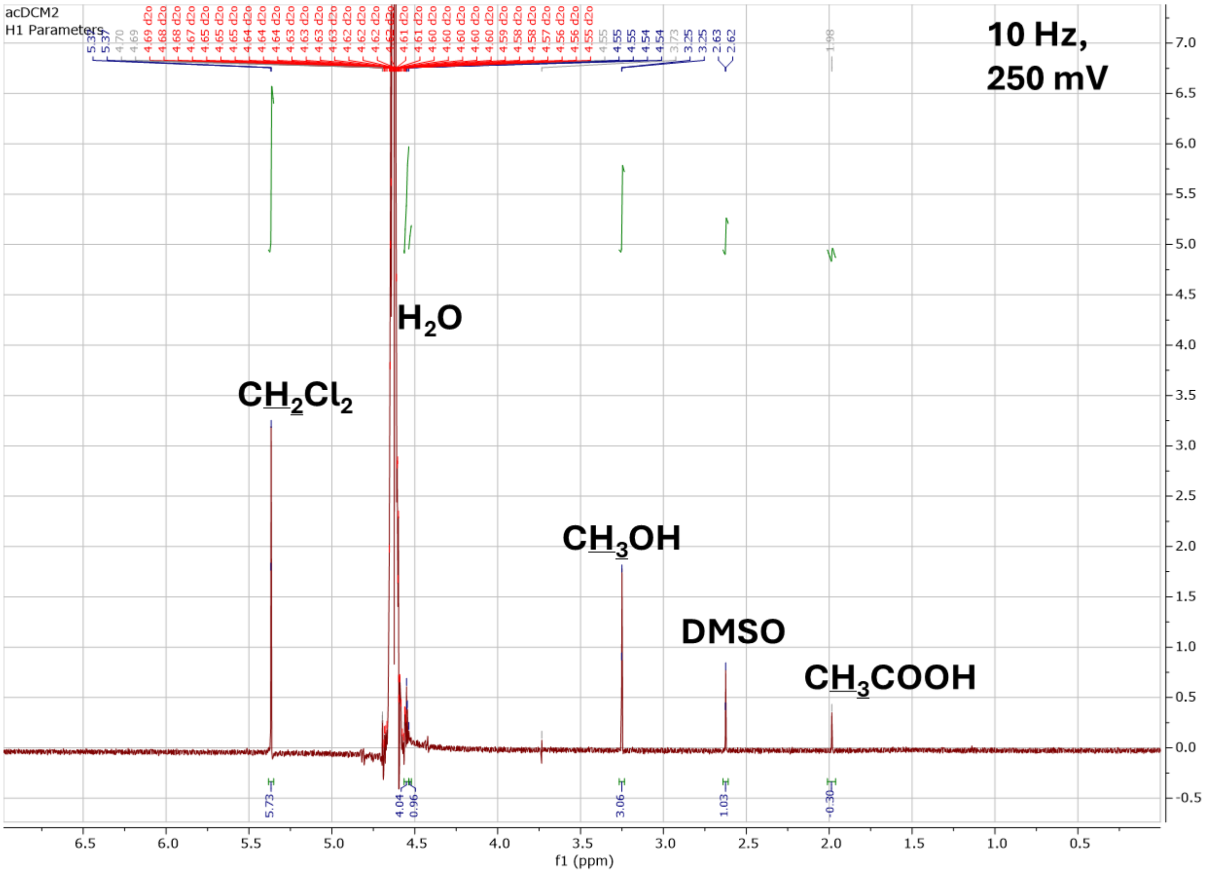

Figure S3. ^{1}H-NMR identification of methane oxidation products generated under the condition of 10 Hz/± 250 mV.

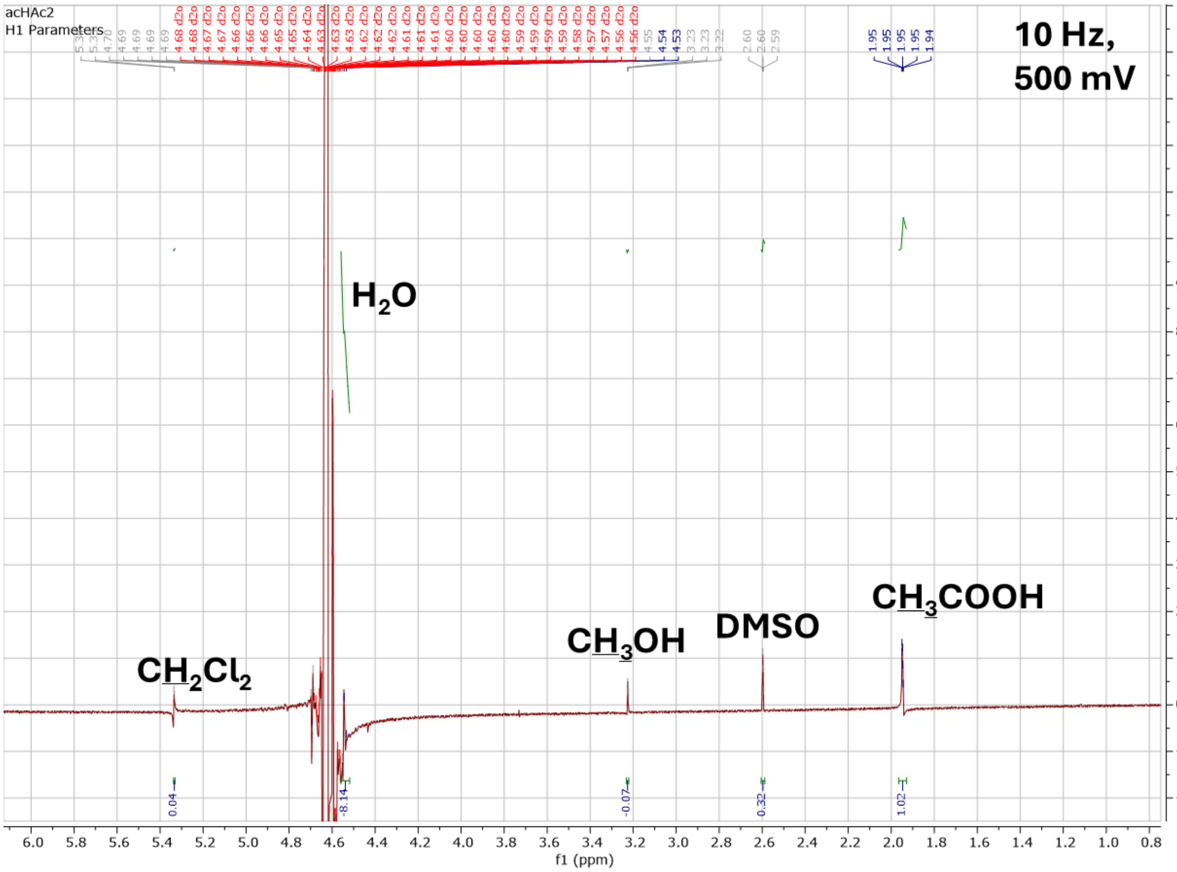

Figure S4. ^{1}H-NMR identification of methane oxidation products generated under the condition of 10 Hz/± 500 mV.

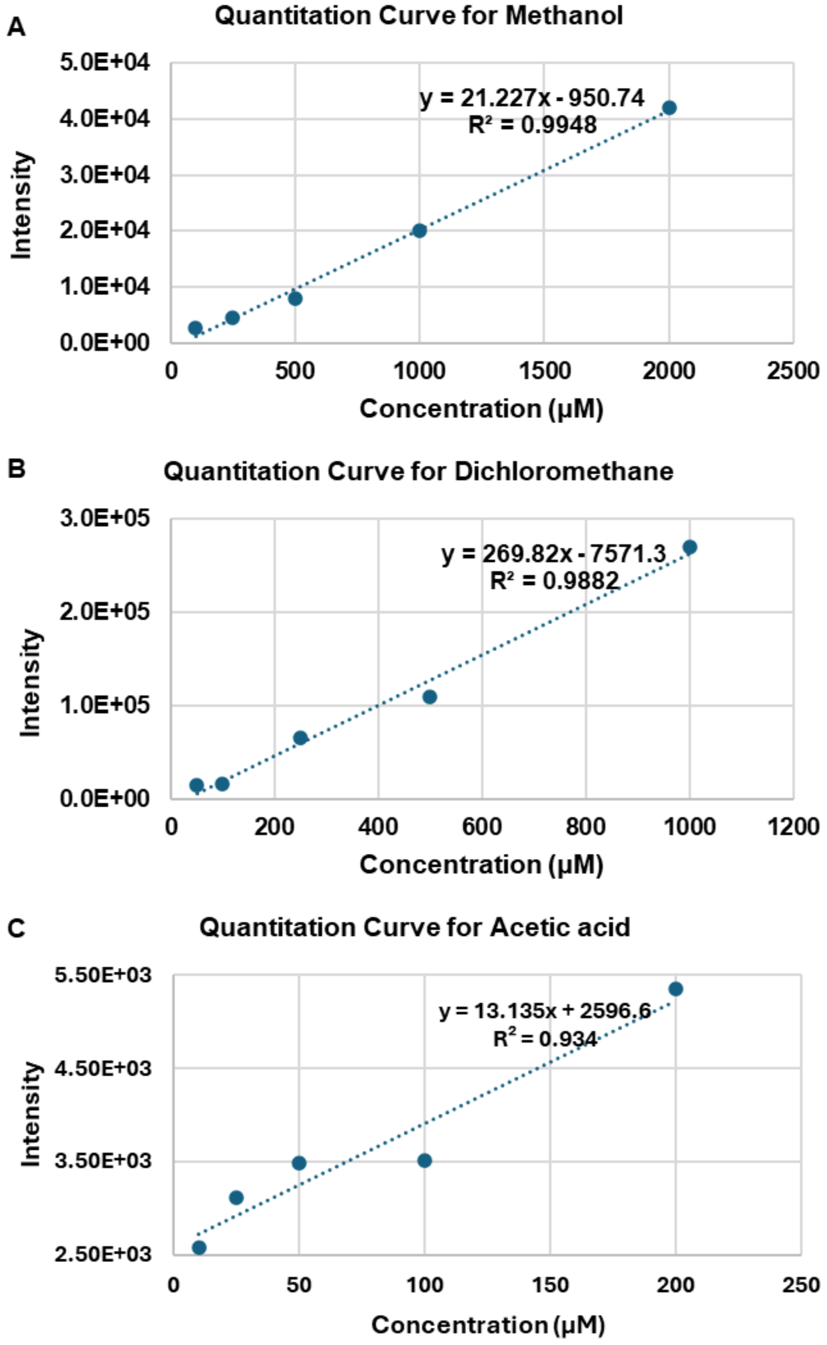

Figure S5. Quantitation curves for determining the concentration of methanol, dichloromethane, and acetic acid generated from the methane oxidation.

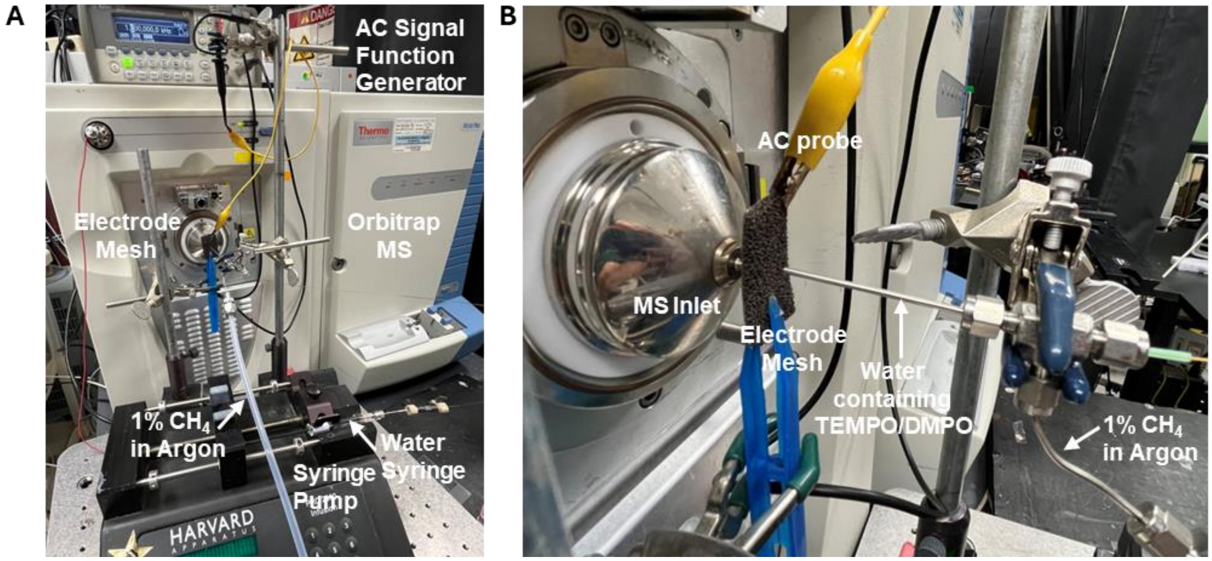

Figure S6. Photos of the (A) on-line setup for the methane oxidation and radicals monitoring by high-resolution mass spectrometer. (B) the zoom-in photo of the on-line reactor composed CuO mesh electrode precoated with salt precipitation and an AC probe and a nebulizing sprayer.

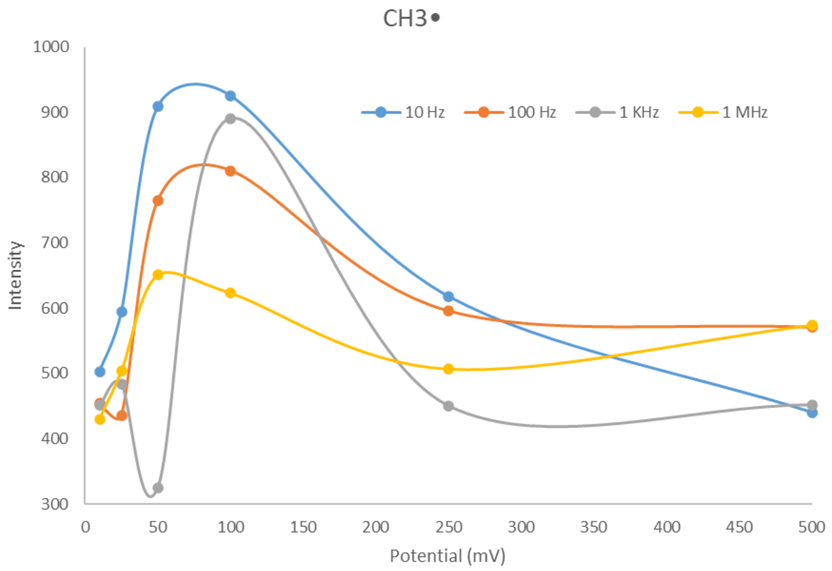

Figure S7. Abundance changes of methyl radical with the applied AC potential under different frequencies.

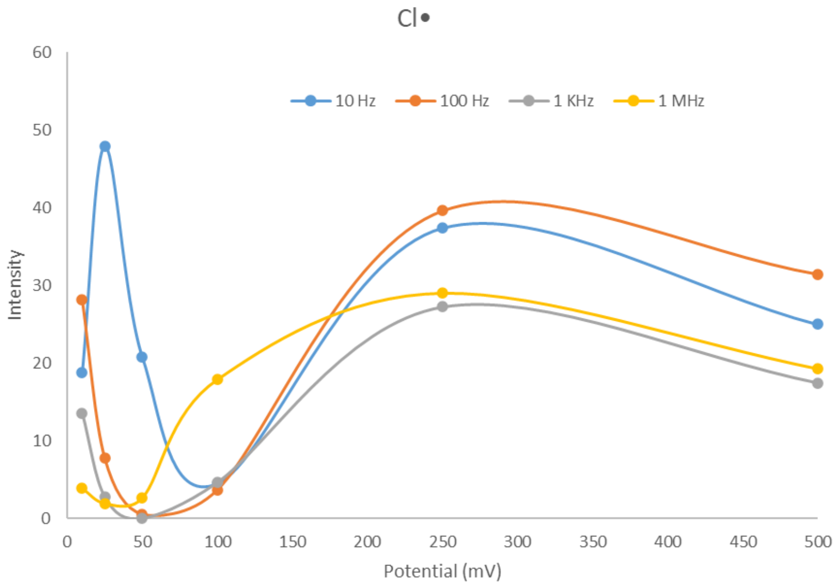

Figure S8. Abundance changes of chlorine radical with the applied AC potential under different frequencies.

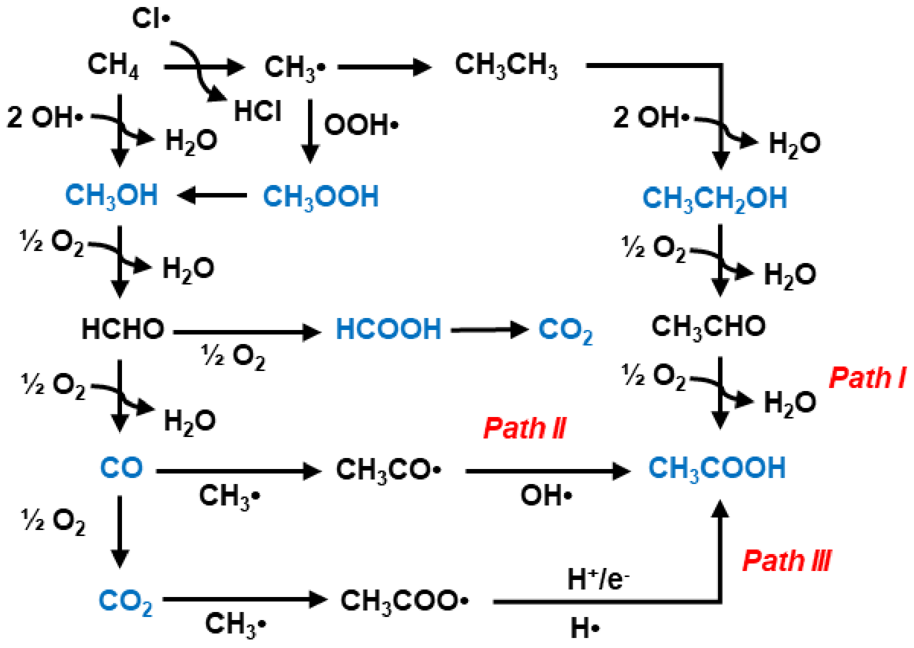

Figure S9. Proposed pathways of the acetic acid formation derived from CO, CO_2, and C_2H_6 respectively.

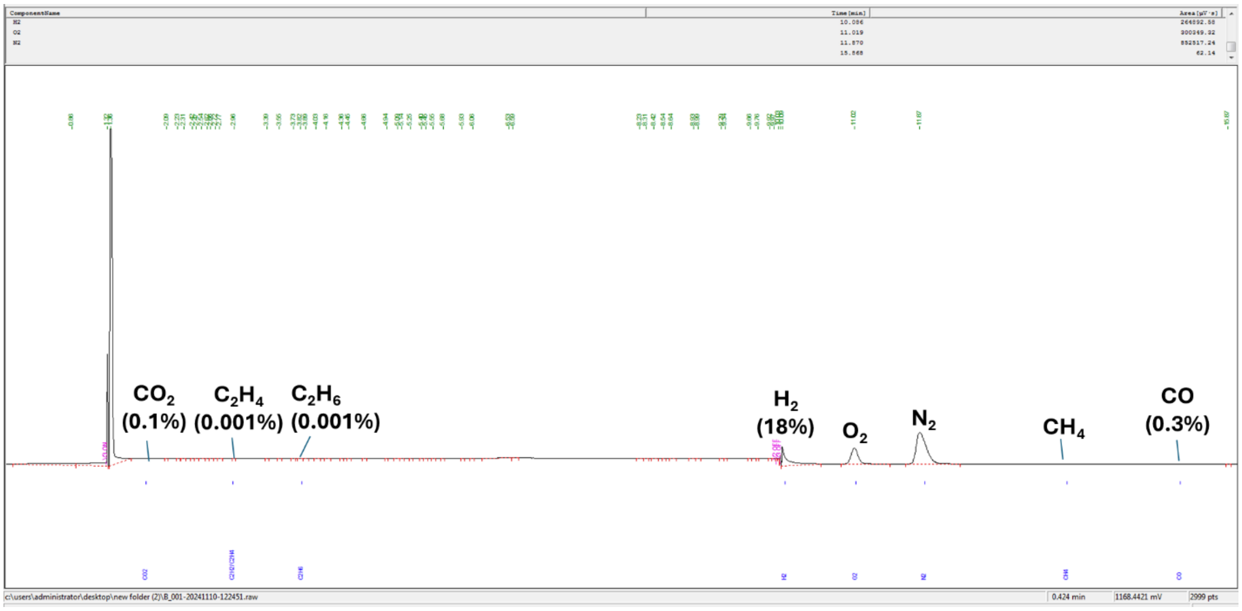

Figure S10. Gas chromatography detection result for the partial oxidation of 1% methane gas in the air after being micro-bubbled into saltwater applied with ± 100 mV alternating potential at a frequency of 10 Hz.

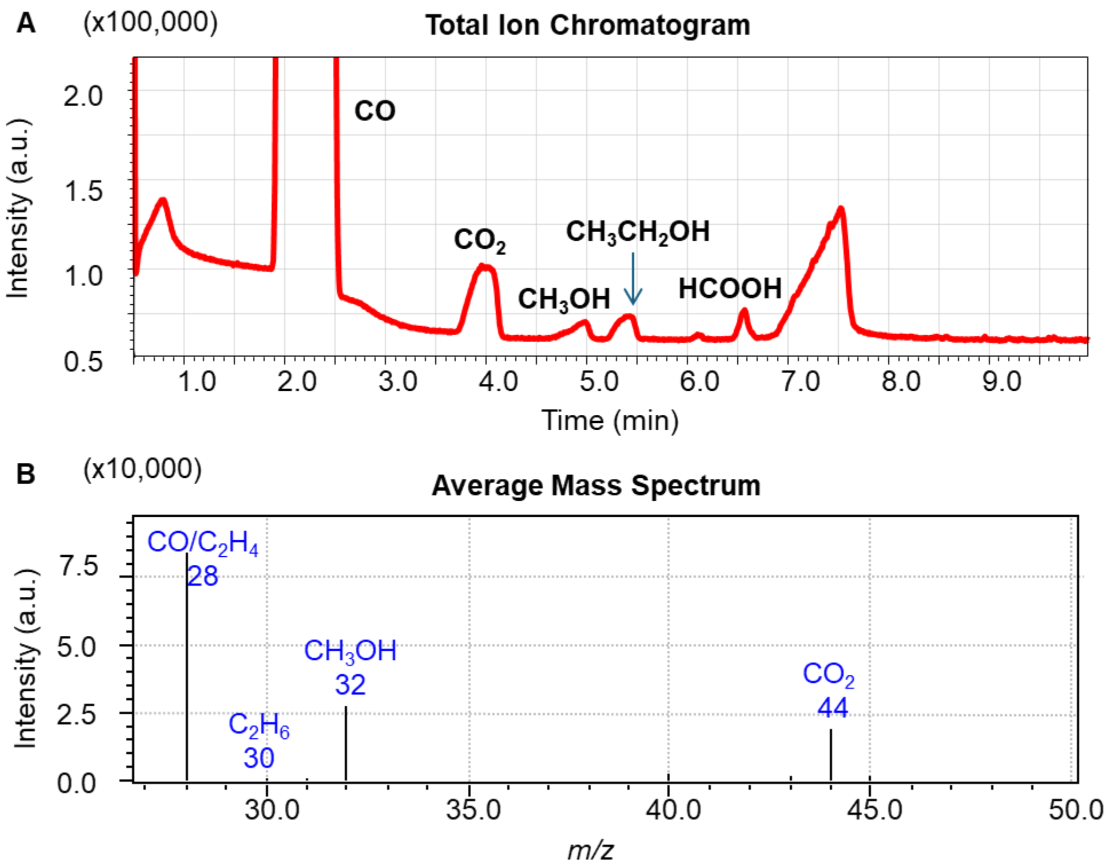

Figure S11. Gas products detection from partial oxidation of methane by gas chromatograph-mass spectrometry. The components were characterized by (A) total ion chromatogram and (B) average mass spectrum.

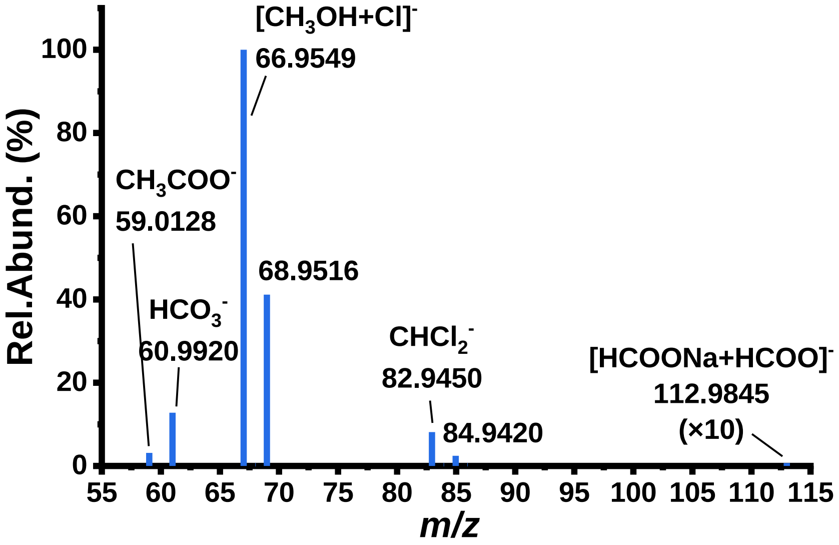

Figure S12. Typical mass spectrum of partial oxidation of methane that is micro-bubbled through saltwater under the conditions of ± 100 mV and 10 Hz. The formic acid was detected in a sodiated dimer form with trace amount.

Table S1. Performance comparison with different catalytic oxidation of methane into methanol.

Method	Catalyst	Condition	Selectivity	Conversion Rate	Ref.
This work	CuO@Cu foam	AC 0.1 V, 10 Hz, 25 °C, 3% KCl (NaCl)	90 %	887 µM h^{-1}	
electrocatalysis	CoO_x	0.1 M K2CO3, pH 11, 17 °C, 0.5~1.0 V vs SHE	70-80%	50 µM h^{-1}	1
electrocatalysis	V_2O_5	[BMIM]BF4 electrolyte -1.45 V vs Ag/AgCl	60-80%	352.5 µmol g^{-1} h^{-1}	2
Electrothermal catalysis	Carbon cloth electrode	phosphotungstic acid, -0.2 V vs Ag/AgCl	74.3%	19.9 µmol g^{-1} h^{-1}	3
Contact Electro Catalysis	PTFE nanoparticles	25 °C, 40 KHz, 200 W ultrasound	34%	151.2 µmol g^{-1} h^{-1}	4
Photocatalysis	Ag/InGaN	200-800 nm	93%	25 µmol cm^{-2} h^{-1}	5
Photocatalysis	Pt/TiO2	10 bar, 25 °C 200-800 nm	70%	14 µmol g^{-1} h^{-1}	6
Photocatalysis	1.0%$AuPd_{0.5}$/ZnO	30 bar, 25 °C 200-800 nm	88.2%	81.0 µmol h^{-1}	7
Photocatalysis	0.75% $Au_{0.5}$/ZnO	5-15 bar, 30 °C	99.1%	5 µmol h^{-1}	8
Photocatalysis	$Au_{0.2}Cu_{0.15}$/ZnO	20 bar, 25 °C 365 nm	80%	120 µmol h^{-1}	9
Thermocatalysis	NU-1000 metal−organic framework (MOF)	150 °C, 1 bar	45-60%	60 µmol g^{-1} h^{-1}	10
Thermocatalysis	AuPd/zeolite crystals	70 °C	92%	91.6 µmol mg^{-1} h^{-1}	11
Thermocatalysis	Cu in zeolite	He (400 °C), 7 bars H2O (200 °C)	97%	1.6 µmol mg^{-1} h^{-1}	12

Reference

[1] Shen, K., Kumari, S., Huang, Y.C., Jang, J., Sautet, P. and Morales-Guio, C.G., 2023. Electrochemical oxidation of methane to methanol on electrodeposited transition metal oxides. *Journal of the American Chemical Society*, *145*, 6927-6943.

[2] Jiang, H., Zhang, L., Wang, Z., Han, Z., Ma, L., Li, A., Sun, Y., Tang, Y., Wan, P., Zhang, R. and Chen, Y., 2023. Electrocatalytic methane direct conversion to methanol in electrolyte of ionic liquid. *Electrochimica Acta*, *445*, p.142065.

[3] Chang, J., Wang, S., Hülsey, M.J., Zhang, S., Lou, S.N., Ma, X. and Yan, N., Electrothermal Conversion of Methane to Methanol at Room Temperature with Phosphotungstic Acid. *Angewandte Chemie International Edition*, p.e202417251.

[4] Li, W., Sun, J., Wang, M., Xu, J., Wang, Y., Yang, L., Yan, R., He, H., Wang, S., Deng, W.Q. and Tian, Z.Q., 2024. Contact-Electro-Catalysis for Direct Oxidation of Methane under Ambient Conditions. *Angewandte Chemie*, *136*(20), p.e202403114.

[5] Zhou, P., Tang, S., Ye, Z., Navid, I.A., Xiao, Y., Sun, K. and Mi, Z., 2024. Water-promoted selective photocatalytic methane oxidation for methanol production. *Chemical Science*, *15*(4), pp.1505-1510.

[6] Ta, Q.T.H., Tran, D.L., Hoang, N.T., Nguyen, P.K.T. and Nguyen, D.H., 2024. Highly selectivity of methanol production from methane oxidation over Pt-modified TiO2 photocatalyst. *Materials Today Communications*, *40*, p.110187.

[7] Zhou, Q., Tan, X., Wang, X., Zhang, Q., Qi, C., Yang, H., He, Z., Xing, T., Wang, M., Wu, M. and Wu, W., 2024. Selective Photocatalytic Oxidation of Methane to Methanol by Constructing a Rapid O2 Conversion Pathway over Au–Pd/ZnO. *ACS Catalysis*, *14*(2), pp.955-964.

[8] Zhou, W.; Qiu, X.; Jiang, Y.; Fan, Y.; Wei, S.; Han, D.; Niu, L.; Tang, Z. Highly selective aerobic oxidation of methane to methanol over gold decorated zinc oxide via photocatalysis. J. Mater. Chem. A 2020, 8, 13277−13284.

[9] Luo, L., Gong, Z., Xu, Y., Ma, J., Liu, H., Xing, J. and Tang, J., 2021. Binary Au–Cu reaction sites decorated ZnO for selective methane oxidation to C1 oxygenates with nearly 100% selectivity at room temperature. *Journal of the American Chemical Society*, *144*(2), pp.740-750.

[10] Ikuno, T., Zheng, J., Vjunov, A., Sanchez-Sanchez, M., Ortuño, M.A., Pahls, D.R., Fulton, J.L., Camaioni, D.M., Li, Z., Ray, D. and Mehdi, B.L., 2017. Methane oxidation to methanol catalyzed by Cu-oxo clusters stabilized in NU-1000 metal–organic framework. *Journal of the American Chemical Society*, *139*(30), pp.10294-10301.

[11] Jin, Z., Wang, L., Zuidema, E., Mondal, K., Zhang, M., Zhang, J., Wang, C., Meng, X., Yang, H., Mesters, C. and Xiao, F.S., 2020. Hydrophobic zeolite modification for in situ peroxide formation in methane oxidation to methanol. *Science*, *367*(6474), pp.193-197.

[12] Sushkevich, V.L., Palagin, D., Ranocchiari, M. and Van Bokhoven, J.A., 2017. Selective anaerobic oxidation of methane enables direct synthesis of methanol. *Science*, *356*(6337), pp.523-527.
